# Automated assessment of cardiac pathologies on cardiac MRI using T1-mapping and late gadolinium phase sensitive inversion recovery sequences with deep learning

**DOI:** 10.1186/s12880-024-01217-4

**Published:** 2024-02-13

**Authors:** Aleksandra M. Paciorek, Claudio E. von Schacky, Sarah C. Foreman, Felix G. Gassert, Florian T. Gassert, Jan S. Kirschke, Karl-Ludwig Laugwitz, Tobias Geith, Martin Hadamitzky, Jonathan Nadjiri

**Affiliations:** 1 Interventional Radiology, School of Medicine & Klinikum rechts der Isar, Technical University of Munich, Ismaninger Str. 22, 81675 Munich, Germany; 2 Department of Diagnostic and Interventional Radiology, School of Medicine & Klinikum rechts der Isar, Technical University of Munich, Ismaninger Str. 22, 81675 Munich, Germany; 3 TUM-Neuroimaging Center, School of Medicine & Klinikum rechts der Isar, Technical University of Munich, Ismaninger Str. 22, 81675 Munich, Germany; 4 Department of Medicine I, School of Medicine & Klinikum rechts der Isar, Technical University of Munich, Ismaninger Str. 22, 81675 Munich, Germany; 5 Department of Radiology, German Heart Center Munich, Technical University of Munich, Lazarettstraße 36, 80636 Munich, Germany

**Keywords:** Magnetic resonance imaging, Deep learning, Classification, Cardiomyopathies

## Abstract

**Background:**

A deep learning (DL) model that automatically detects cardiac pathologies on cardiac MRI may help streamline the diagnostic workflow. To develop a DL model to detect cardiac pathologies on cardiac MRI T1-mapping and late gadolinium phase sensitive inversion recovery (PSIR) sequences were used.

**Methods:**

Subjects in this study were either diagnosed with cardiac pathology (*n* = 137) including acute and chronic myocardial infarction, myocarditis, dilated cardiomyopathy, and hypertrophic cardiomyopathy or classified as normal (*n* = 63). Cardiac MR imaging included T1-mapping and PSIR sequences. Subjects were split 65/15/20% for training, validation, and hold-out testing. The DL models were based on an ImageNet pretrained DenseNet-161 and implemented using PyTorch and fastai. Data augmentation with random rotation and mixup was applied. Categorical cross entropy was used as the loss function with a cyclic learning rate (1e-3). DL models for both sequences were developed separately using similar training parameters. The final model was chosen based on its performance on the validation set. Gradient-weighted class activation maps (Grad-CAMs) visualized the decision-making process of the DL model.

**Results:**

The DL model achieved a sensitivity, specificity, and accuracy of 100%, 38%, and 88% on PSIR images and 78%, 54%, and 70% on T1-mapping images. Grad-CAMs demonstrated that the DL model focused its attention on myocardium and cardiac pathology when evaluating MR images.

**Conclusions:**

The developed DL models were able to reliably detect cardiac pathologies on cardiac MR images. The diagnostic performance of T1 mapping alone is particularly of note since it does not require a contrast agent and can be acquired quickly.

## Background

### Introduction

The leading cause of death worldwide persists to be of cardiac origin, accounting for the highest component of European healthcare costs at 200 billion euros annually. The impact of cardiovascular diseases on public health remains concerning, as they were responsible for roughly 85 million disability-adjusted life years in 2019. These circumstances stress the importance of heart disease preventive procedures [1]. Correct and early diagnosis is essential for reducing mortality and severe health consequences [2]. It is challenging for clinicians to detect cardiac pathologies in an early stage before their actual occurrence.

Noninvasive imaging helps in early and reliable heart disease detection [3]. In clinical routine, MRI is the preferred imaging modality for cardiovascular assessment [3, 4]. Remarkable benefits of cardiovascular magnetic resonance (CMR) are the precise characterization of myocardial tissue composition and visualization of underlying pathological processes, which are notably helpful in detecting early changes [3, 5]. CMR has become widely accessible, and its clinical application has grown significantly over the past decades [5, 6]. The increased clinical use of MRI and the constant need for heart disease prevention justify the demand for automated diagnostic tools to efficiently detect the prevalence of heart diseases. Deep learning (DL) represents a promising approach to assist clinicians and streamline their workflow for a faster and more accurate diagnosis [6].

DL inherently learns intrinsic hierarchical data representation instead of handcrafted feature extraction of machine learning algorithms [7, 8]. In recent years convolutional neural networks (CNNs), a type of DL model, gained much popularity for computer vision tasks and are now commonly used for medical image analysis [8]. DL repeatedly achieved state-of-the-art segmentation and classification performance. Some of the best approaches score equal to clinical experts [9, 10]. The most frequent DL applications in CMR analysis are segmentation of heart structures, image acquisition improvement, and automated assessment from cine images. However, there is less literature evaluating CNN-based diagnosis of cardiomyopathies from late gadolinium enhancement (LGE) or mapping images [6, 11].

LGE is an established technique in clinical practice and LGE patterns on MR images play an essential role in diagnosing cardiomyopathies and guiding therapy [5, 12, 13]. The presence and distribution of contrast agent can reveal focal pathologic changes in the myocardium, such as necrosis, fibrosis, amyloid deposition, and edema, with high spatial resolution [3, 5]. In addition, the phase-sensitive inversion recovery (PSIR) sequence acts as an inversion time optimizer and increases the robustness of LGE examinations [14].

A new MRI approach, called mapping, allows assessing pathologic areas by visualizing basic tissue magnetization properties [5, 15]. T1 relaxation time increases in fibrosis, amyloidosis, and edema [3, 5]. Detection of diffuse diseases is a significant advantage of mapping as diffuse fibrosis is often occult and may be absent on LGE images and other imaging techniques [5, 15]. Growing evidence reasserts the diagnostic and preventive value of T1 mapping for contrast agent- and radiation-free screening of the hearth [4]. The downsides of this method are limited spatial resolution, lack of universal reference values, and dependency of relaxation time on MRI field strength and protocol, which restrains reproducibility [3, 16].

Automated diagnostic systems like CNNs can help professionals detect diseases early in a more accurate way that is less time-consuming and costly [2, 17]. In particular, inexperienced physicians can benefit from a reference finding in their decision-making process [12].

Considering the benefits of deep learning and the need for affordable high-volume screening methods, we applied a DL network to detect cardiac diseases. This study aimed to evaluate a CNN model’s capability to classify pathologic and normal myocardium on LGE PSIR and T1 mapping images. All used abbreviations can be seen in Table 1 at the end of the paper.

**Table 1:** Abbreviations

Abbreviation	Description
CMR	Cardiovascular Magnetic Resonance
DL	Deep learning
CNN	Convolutional Neural Network
LGE	late gadolinium enhancement
PSIR	phase-sensitive inversion recovery
DCM	dilated cardiomyopathy
HCM	hypertrophic cardiomyopathy
Grad-CAM	Gradient-weighted class activation map
EF	ejection fraction

### Comparison with previous studies

Compared to our approach, previous studies assessing cardiac diseases on CMR used different DL techniques and MRI sequences [8, 18, 19]. Frequently used DL applications in CMR automated assessment are based on volumetric features from the prior segmentation mask of heart structures, especially from cine images. However, there is less literature evaluating CNN-based diagnosis of cardiomyopathies from late gadolinium enhancement (LGE) or mapping images [6, 11]. In addition, only a few studies solely use image-based features, with most of them focusing on a specific cardiac disease.

We opted to explore less commonly used CMR sequences in automated assessment studies, such as T1 mapping and LGE. Additionally, we attempted to train our model under realistic clinical conditions with an unbalanced dataset and a diverse range of cardiac pathologies. A limitation of our approach is its binary classification, differentiating only between normal and abnormal cases, in contrast to multiclass classifications. The differentiation between specific pathologies would be a subsequent project benefiting from our diverse dataset.

The studies selected for comparison with our approach are outlined in Table 2. The ACDC dataset contains 150 samples with a balanced distribution of 5 cardiac diagnoses [8, 18, 19]. Similar to our approach, it doesn’t solely focus on one single pathology. Despite dealing with an imbalanced dataset, we incorporated a broader spectrum of 16 cardiac diseases. This approach reflects a more real-life clinical scenario. The three studies working with the ACDC challenge dataset [8, 18, 19] represent multiclass classification based on combined automated segmentation and classification on cine CMR. Thus, the diagnosis relies on calculated cardiac parameters like ejection fraction or ventricle volumes from the segmentation masks. In contrast, our model solely uses image-based features from different sequences. Moreover, our study included all planes, whereas the ACDC dataset consists of short-axis views only. We also tested two different MRI sequences to compare their performance. Additionally, of the three ACDC studies mentioned, only ML methods obtained state-of-the-art classification results compared to DL.

**Table 2:** Comparison of previous studies

Research group	MRI sequence	AI method for classification	Dataset	AI task	Acc (%)	AUC
Zhang et al. [23]	Nonenhanced cine	Fully connected discriminative network (DL)	299 patients (MI: *n* = 212)	Detecting and delineating chronic MI. Classification as normal or infarcted myocardium.	-	0.94
Snaauw et al. [18]	Cine	DenseNet (DL)	ACDC (DC, HCM, MI, ARV, NOR)	End-to-end diagnosis and segmentation.	78	-
Khened et al. [19]	Cine	Random forest method (ML)	ACDC (DC, HCM, MI, ARV, NOR)	Fully automated segmentation and classification.	90	-
Ammar et al. [8]	Cine	Classifier ensemble combining multilayer perceptron, random forest, and support vector machine (ML)	ACDC (DC, HCM, MI, ARV, NOR)	Automated pipeline for segmentation and prediction.	92	-
Agibetov et al. [20]	LGE Cine T1 mapping	VGG16 CNN pretrained on ImageNet (DL)	502 patients (CA: *n* = 82)	Detection of potential patterns of CA.	94	0.96
Ohta et al. [12]	MDE	GoogLeNet AlexNet ResNet-152 CNNs (DL)	200 patients	Detection and classification of MDE patterns.	78.9 to 82.1	0.938 to 0.948
Martini et al. [21]	LGE	3 pretrained CNNs (DL), Comparison to gradient boosting classifier (ML)	206 patients with suspected CA	Automated classification as amyloidosis present or absent based on average probability from the 3 CNNs.	88 (DL) 90 (ML)	0.982 (DL) 0.952 (ML)
El-Rewaidy et al. [22]	Native T1 mapping	Linear support vector machine and regression model (ML)	321 patients (Control, HCM, DCM)	Texture analyses on myocardial native T1 mapping to differentiate between fibrosis patterns in patients with HCM and DCM.	89.3	-

Note. — MI = myocardial infarction, HCM = hypertrophic cardiomyopathy, DCM = dilated cardiomyopathy, CA = cardiac amyloidosis, ARV = abnormal right ventricle, NOR = normal, LGE = late gadolinium enhancement, MDE = myocardial delayed enhancement, DL = deep learning, ML = machine learning

Noteworthy is the ACDC dataset’s exclusion of ambiguous cases with diagnostic boundary values for handcrafted features [18]. This may potentially affect the performance of the ML methods. In contrast, our dataset also contained adversarial examples near the boundary of the two classes. This contributes to an improved ability to detect diseases, offering a more accurate, time-efficient, and cost-effective diagnostic approach.

Agibetov et al. [20] and Martini et al. [21] focused on one specific cardiac disease, namely amyloidosis, and performed a binary classification. Both integrated different cardiac diseases into their control groups. Both studies represent an unbalanced dataset, mirroring a more realistic prevalence, as we aimed for in our study. The 82 amyloidosis cases in Agibetov et al. [20] were all in advanced stages, raising concerns about the model’s capacity to detect early stages. Unlike Martini et al. [21], who did not integrate PSIR with LGE sequences, we enhanced the robustness of the LGE examination by combining LGE with PSIR.

Ohta et al. [12] cropped image regions outside the heart, risking information loss. In contrast, our model was trained on whole images. This allows the recognition of misdiagnosis originating from false attention points in different organs or structures. Moreover, Ohta et al. [12] focused solely on detecting MDE patterns without detailed pathological diagnoses. Pattern classification was performed slice-wise and not case-wise. This could be problematic for the final diagnosis, as patients do not necessarily show the same MDE pattern in each slice. Our study, on the other hand, classifies the entire case by stacking 10 slices per subject.

The ML network of El-Rewaidy et al. [22] was trained on one single CMR sequence. Although they performed multiclass classifications, their dataset contains only two different cardiac pathologies next to normal cases. Hence, not fully representing a realistic clinical prevalence. In contrast, our study compares the performance of two distinct sequences, LGE and T1 mapping, with a more diverse dataset of cardiac pathologies.

Finally, all the above-mentioned studies share the characteristic of being conducted at a single center with a single vendor, lacking external validation. While each referenced study contributes valuable insights, our study stands out in its performance comparison of two less frequently evaluated CMR sequences. The exceptional diversity in our dataset, containing 16 different cardiac pathologies, mirrors realistic daily clinical conditions. This approach contributes to a more robust automated assessment system for cardiac diagnoses.

## Materials and methods

### Study design

This retrospective, single-center study was approved by our local ethics committee. All authors approved the manuscript and submission. No industry support was received. Our approach aimed to develop a DL model that automatically detects cardiac pathologies on CMR and helps streamline the diagnostic workflow.

### Data

MR images obtained from consecutive examinations were selected from the picture archiving and communication system of the German Heart Center Munich. All performed examinations had a clinical indication. For reference, we incorporated a control group with normal myocardium, as declared by report. Images were analyzed by two Level III CMR readers (certified by the European Association of Cardiovascular Imaging) and documented in binary representation by consent. Diagnostic criteria for both groups, normal and pathologic myocardium, were based on established guidelines in the clinical routine. All diagnoses were made with final consensus and agreement of the department of Cardiology at daily conferences. All used data underwent an anonymization process.

The 1.5 Tesla MRI scanner, Magnetom Avanto Siemens, was used for image acquisition. CMR was conducted following the methodology previously outlined in reference [24]. In both pre- and post-contrast T1 mapping, we utilized a Modified-Look-Locker-Inversion-Recovery (MOLLI) prototype sequence (Siemens WIP 780B) with 3 inversion pulses and adhered to the 4-(1)-3-(1)-2 readout pattern, as outlined in Kellmann et al.‘s publication [25]. Further parameters included Field of View (FOV: 224 × 279 mm2) and slice thickness (8 mm). MOLLI T1 mapping involved capturing IR measurements in a single breath-hold, incorporating motion correction, and the reconstruction of T1 maps. This process was integrated as an in-line function within the MRI scanner. To calculate the ECV, we performed another T1 mapping 10 min after contrast administration. LGE evaluation took place 15 min after the administration of the contrast agent, using a T1-weighted inversion recovery gradient echo sequence. 15 min after the contrast agent was given, we conducted a LGE assessment utilizing a T1-weighted inversion recovery gradient echo sequence. To nullify the signal from normal myocardium the inversion time was individually adjusted. The pulse sequence parameters included a Field of View (FOV) of 340 × 276 mm², Echo Time (TE) of 3.37 ms, Repetition Time (TR) of 6.0 ms, an 8 mm slice thickness, a flip angle of 30°, and excitation occurring every second heartbeat. Contiguous short-axis slices covering the entire left ventricle from its base to its apex, along with a four-chamber view of the left ventricle were obtained in all acquisitions [24].

### Data preprocessing

Extracted data images were stored and preprocessed in the Digital Imaging and Communications in Medicine (DICOM) format for all datasets. To cover the whole heart ten slices at different levels were stacked for each subject. We resized images to 224 × 224 pixels to uniformize spatial dimension over the dataset since the pretrained neural network architecture only accepted inputs of the same size. Adjacent areas outside the cardiac region were not cropped. Therefore, representing realistic conditions of a routinely acquired CMR scan that covers different surrounding structures of the heart. In addition, we performed an image normalization operation. Adjusting the intensity level of all pixels to the range of 0 to 1 resulted in an independent and homogenous intensity distribution. All steps were conducted slice by slice. Before transferring the data into the CNN model, the data was converted to the Joint Photographic Experts Group (JPEG) format. No further image modifications or adjustments were implemented.

### Data partition

Subjects were split 65/15/20% for training, validation, and hold-out testing. As our dataset presents an imbalanced-class distribution, we applied stratified sampling for dividing the data. In this manner, the sets are disjoint at patient level, reducing the probability of creating a bias.

### DenseNet model

The DL model was based on an ImageNet [26] pretrained DenseNet-161 and implemented using PyTorch [27] and fastai [28] libraries.

DenseNet-161 [29] is a CNN type characterized by a dense connectivity pattern that allows for a deeper architecture than previous CNN models without performance degradation. The connectivity pattern consists of additional direct connections from any layer to all subsequent layers. This improves the information flow through the network, helping to alleviate the vanishing-gradient problem that occurs as networks grow larger. By feature map concatenation instead of summation, all preceding maps are accessible anytime for every layer. A more accurate internal representation is reached. Therefore, the final classifier can make a decision based on all collected feature maps. In addition, the model reuses parameters, eliminating the need to relearn redundant features [29].

Huang et al. [29] showed that on benchmark datasets like ImageNet, DenseNet outperforms other state-of-the-art CNN models or shows comparable performance using fewer parameters and less computation power and time. In particular, DenseNet-161 showed good performance [29]. Therefore, in our study we propose using the DenseNet-161 model with a depth of 161 layers. For more detailed information on the DenseNet-161 architecture refer to [29].

### Modifications to the model

The architecture of DenseNet-161 was barely modified. To fit the binary classification task, we revised the classification layer from 1000D fully connected to 2D fully connected. No further changes were made to the original architecture of the ImageNet pretrained DenseNet-161 model.

### Initialization of model parameters

We used a version of DenseNet-161 with already pretrained weights on ImageNet, available through torchvision [30]. ImageNet is a labeled database with millions of natural images for training and validation [26]. It is a benchmark for visual recognition tasks and is widely used for medical image analysis based on transfer learning [26, 31]. Transfer learning compensates for one of the main challenges in deep learning, which is the lack of labeled medical data. It also provides a better starting point than randomly initialized weights [31–33].

### Training and hyperparameters

The datasets for both models were from the same group of patients and differed only in the selected sequences. The DL models for T1-mapping and PSIR sequences were developed separately using similar training parameters. The batch size was set to 32 and the epochs number to 8. We used weight decay and stochastic gradient descent with Momentum for weight optimization. Categorical cross entropy was used as the loss function with a cyclic learning rate (1e-3). Based on the performances on the validation set, we selected the final model for the hold-out testing.

### Augmentation

We applied random rotation and mixup to augment our training images. Random rotation involves rotating an image by a random angle, enhancing model robustness to different object orientations. Mixup blends two random pairs of images and their corresponding labels, creating new training samples. The main idea is to artificially enlarge the data and increase the diversity of samples. This allows the model to learn features independent of their location and orientation in the image [33–35].

Zhang et al. [36] show that the mixup strategy improves the robustness to adversarial noise, such as artifacts and various signal-to-noise ratios in medical images. Therefore enhances the generalization capability of deep neural networks.

### Performance evaluation metrics

In our study, we opted for commonly employed evaluation metrics in ML and DL image classification research, aligning with established practices in the scientific community. In addition, doctors and other healthcare professionals involved in creating AI models must grasp the potential enhancements these models could bring to patient care. Since these metrics often pose challenges in terms of interpretability, we opted for easy-to-understand metrics. This choice facilitates meaningful comparisons with similar studies and effectively communicates the performance of our DL models.

**Fig. 1:**
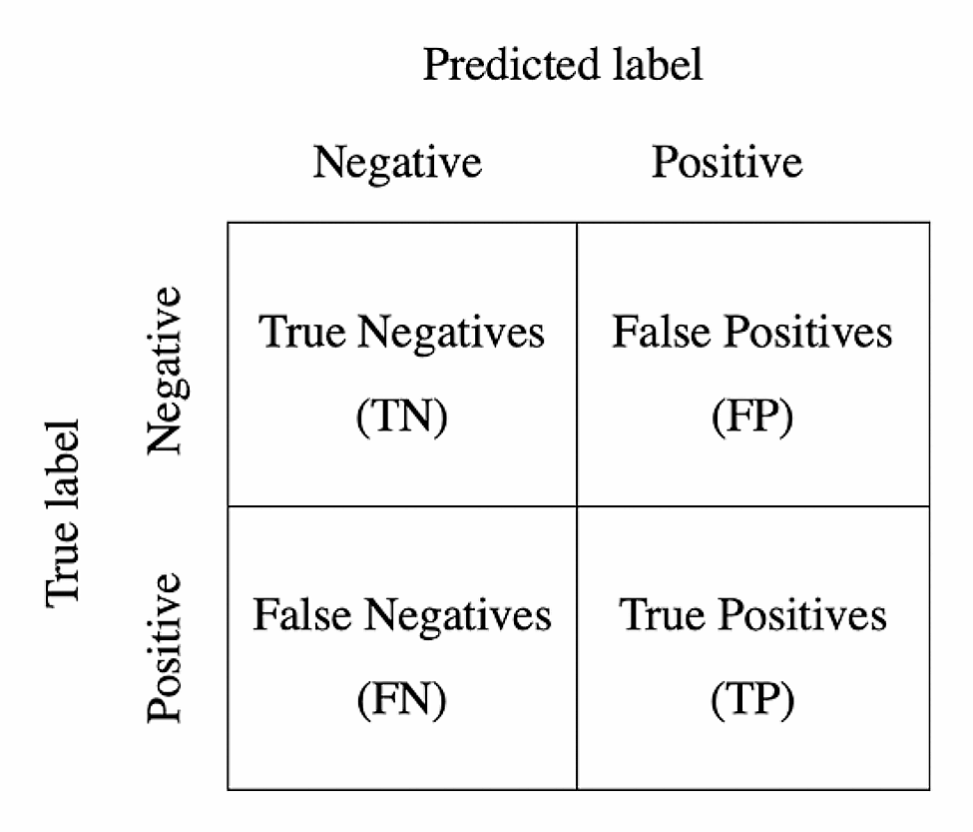
Framework of the confusion matrix for a binary classification

We evaluated the final model performance on the hold-out test set with sensitivity, specificity, accuracy, false positive rate, false negative rate, and confusion matrix. The framework of the confusion matrix for our binary classification task can be seen in Fig. 1. In our case, the negative label represents normal myocardium, and the positive label denotes abnormal myocardium.

Correctly identified cases correspond to True Negatives (TN) and True Positives (TP). Incorrectly predicted classes are shown in Fig. 1 as False Negatives (TN) and False Positives (FP). Thus, the confusion matrix allows for recognizing what kind of errors the model makes.

Sensitivity (Eq. 1) is the percentage of correctly detected abnormal cardiac muscles among all cases of heart disease. In medicine, this metric is crucial as missed myocardial diseases, due to false negatives, can have severe consequences for patient health.

1 $${\rm Sensitivity }=\frac{\rm TP}{\rm TP+FN}$$

Specificity (Eq. 2) is the percentage of cases rightly identified as having a normal heart muscle among all subjects without heart disease.

2 $${\rm Specificity }=\frac{\rm TN}{\rm TN+FP}$$

Accuracy (Eq. 3) is the proportion of all correctly predicted cases of the total number of observations.

3 $${\rm Accuracy }=\frac{\rm TP+TN}{\rm TP+TN+FP+FN}$$

The Receiver operating characteristics (ROC) curve can be used for binary classification tasks. It represents a probability plot of the TP rate, called sensitivity, against the FP rate at various threshold points. Different threshold settings change the sensitivity and specificity and can lower the false negative rate. The curve visualizes the ability of a classifier to discriminate between positive (abnormal) and negative (normal) classes. The ROC curve can be summarized in a single value, called the area under the curve (AUC). The AUC offers a comprehensive assessment of the performance as it effectively captures the algorithm’s capacity to differentiate between positive and negative cases. For example, a network with an AUC of 0.5 is not able to differentiate between two classes Whereas values over 0.5 indicate a chance to distinguish them. In general, the higher the AUC value, the better the model’s predictive accuracy. Thus, the ROC curve and AUC value help validate the model’s ability to diagnose disease and help decide whether to implement the network.

Gradient-weighted class activation maps (Grad-CAMs) visualize the decision-making process of the DL model. The proposed technique produces a class-specific heatmap based on an input image. It highlights regions that the network focuses on while predicting a class of interest. This approach enhances the transparency of the DL algorithm, making the output more explainable. Thus, dataset biases can be identified, and inexperienced users can more easily distinguish between a strong and weak network.

## Results

### Data

We included patients who underwent an CMR between January 2016 to September 2017 with following indications: new and old myocarditis, new and old infarction, cardiological assessment, aortic stenosis, dilated cardiomyopathy (DCM), hypertrophic cardiomyopathy (HCM), myocardial ischemia, storage disease, systemic lupus, cardiomyopathy, muscle dystrophy, pericardial effusion, systemic sclerosis, amyloidosis, Erdheim-Chester disease, and hypereosinophilic syndrome. Of which the most common one was suspected myocarditis.

Stringent image quality criteria were applied for data inclusion and exclusion to ensure the reliability of our findings. Key considerations included adequate resolution, especially for the LGE PSIR sequence. Good contrast and Signal-to-Noise Ratio were crucial for distinguishing normal from abnormal myocardial tissue, leading to the exclusion of images with poor contrast or low Signal-to-Noise Ratio. The absence of significant motion artifacts and proper suppression of blood pool signal in LGE imaging were essential criteria. Images with severe motion artifacts, susceptibility artifacts (e.g. from pacemaker implantation), aliasing artifacts, inadequate blood pool suppression, incomplete coverage, or misalignment between slices were excluded to maintain consistent image quality across slices.

Extracted diagnoses were restricted to acute and chronic myocardial infarction, myocarditis, DCM, HCM, and others. Cardiac diseases recorded under “Others” are listed in Table 3.

**Table 3:** Distribution of cardiac pathologies within the 137 abnormal cases

Pathologies	Number of cases (*n* = 137)
Acute myocardial infarction	10 (7.3)
Chronic myocardial infarction	14 (10.2)
Myocarditis (new and old)	36 (26.3)
Myocarditis new	19 (13.9)
Myocarditis old	17 (12.4)
DCM	19 (13.9)
HCM	6 (4.4)
Others	52 (38.0)
Pericarditis	5 (3.6)
Aortic stenosis	30 (21.9)
Storage disease	2 (1.5)
Systemic sclerosis	4 (2.9)
Cardiomyopathy	3 (2.2)
Amyloidosis	1 (0.7)
Pericardial effusion	2 (1.5)
Unspecific load	3 (2.2)
Exercise-induced myocardial ischemia	1 (0.7)
Arrhythmogenic right ventricular dysplasia	1 (0.7)

Note. — Data are numbers of patients with percentage in parentheses. HCM is hypertrophic cardiomyopathy. DCM is dilated cardiomyopathy.

200 patients consisting of 68 women and 132 men were included. Subjects in our study had a mean age of 53.6 ± 19.9 years. The test set comprises 13 cases classified as normal and 27 cases classified as abnormal, with a total of 40 cases.

A description of our patient’s demographic and clinical characteristics of each partition can be seen in Table 4. Each set represented a similar ejection fraction (EF) and contained more men than women. The prevalence of abnormal cases is overrepresented in all three sets, resulting in an unbalanced dataset with 137 abnormal cases and 63 normal cases. This might cause differences in performance measures.

**Table 4:** Demographic and clinical characteristics of the datasets

Participant characteristics	Training set (*n* = 130)	Validation set (*n* = 30)	Test set (*n* = 40)
Sex			
Women	48 (36.9)	7 (23.3)	13 (32.5)
Men	82 (63.1)	23 (76.7)	27 (67.5)
Baseline EF (%)	57.3 ± 13.3	58.5 ± 12.9	57.9 ± 15.2
No. of normal MRIs	41 (31.5)	9 (30.0)	13 (32.5)
No. of abnormal MRIs	89 (68.5)	21 (70.0)	27 (67.5)
Acute myocardial infarction	6 (6.7)	2 (9.5)	2 (7.4)
Chronic myocardial infarction	11 (12.4)	1 (4.8)	2 (7.4)
Myocarditis (new and old)	24 (27.0)	7 (33.3)	5 (18.5)
DCM	10 (11.2)	4 (19.0)	5 (18.5)
HCM	5 (5.6)	0 (0)	1 (3.7)
Others	33 (37.1)	7 (33.3)	12 (44.4)

Note. — Data are numbers of patients with percentage in parentheses. Ejection fraction (EF) is mean data ± standard deviation. HCM is hypertrophic cardiomyopathy. DCM is dilated cardiomyopathy. Others includes Pericarditis, aortic stenosis, storage disease, systemic sclerosis, cardiomyopathy, amyloidosis, pericardial effusion, unspecific load, exercise-induced myocardial ischemia, arrhythmogenic right ventricular dysplasia. Training, validation, and test set were spilt 65/15/20% retrospectively.

A comparison between the characteristics of all abnormal and normal cases is illustrated in Table 5. No significant differences were found between the groups of normal and abnormal cases. The sex distribution was similar between both classes (men ratio: 65% in normal class vs. 66% in abnormal class). Patients with cardiac disease had a lower EF than the control group (mean EF: 61% ± 0.35% in the normal class vs. 56.6% ± 0.85% in the abnormal class).

Table 3 represents the distribution of cardiac pathologies within the 137 abnormal cases.

**Table 5:** Characteristics of normal and abnormal MRIs per partition

	Normal MRIs (*n* = 63)			Abnormal MRIs (*n* = 137)		
Participant characteristics	Training set (*n* = 41)	Validation set (*n* = 9)	Test set (*n* = 13)	Training set (*n* = 89)	Validation set (*n* = 21)	Test set (*n* = 27)
Sex						
Women	15 (36.6)	2 (22.2)	5 (38.5)	33 (37.1)	5 (23.8)	8 (29.6)
Men	26 (63.4)	7 (77.8)	8 (61.5)	56 (62.9)	16 (76.2)	19 (70.4)
Baseline EF (%)	61.0 ± 6.3	61.3 ± 6.6	60.6 ± 6.1	55.6 ± 15.2	57.3 ± 14.8	56.6 ± 18.0

Note. — Data are numbers of patients with percentage in parentheses. Ejection fraction (EF) data are mean data ± standard deviation.

### Statistical analysis of DL models performances on test sets

The final DenseNet model trained on the PSIR data correctly identified 35 of 40 cases, attaining an overall accuracy of 88%. Whereas the final DenseNet model trained on the T1 mapping data correctly identified 25 of 40 cases, attaining an overall accuracy of 70%.

A comparison of performance metric values is represented in Table 6. The PSIR-based model offers 100% sensitivity, recognizing all abnormal cases at the cost of a higher false positive rate of 63% compared to 46% of the T1 mapping-based model.

**Fig. 2:**
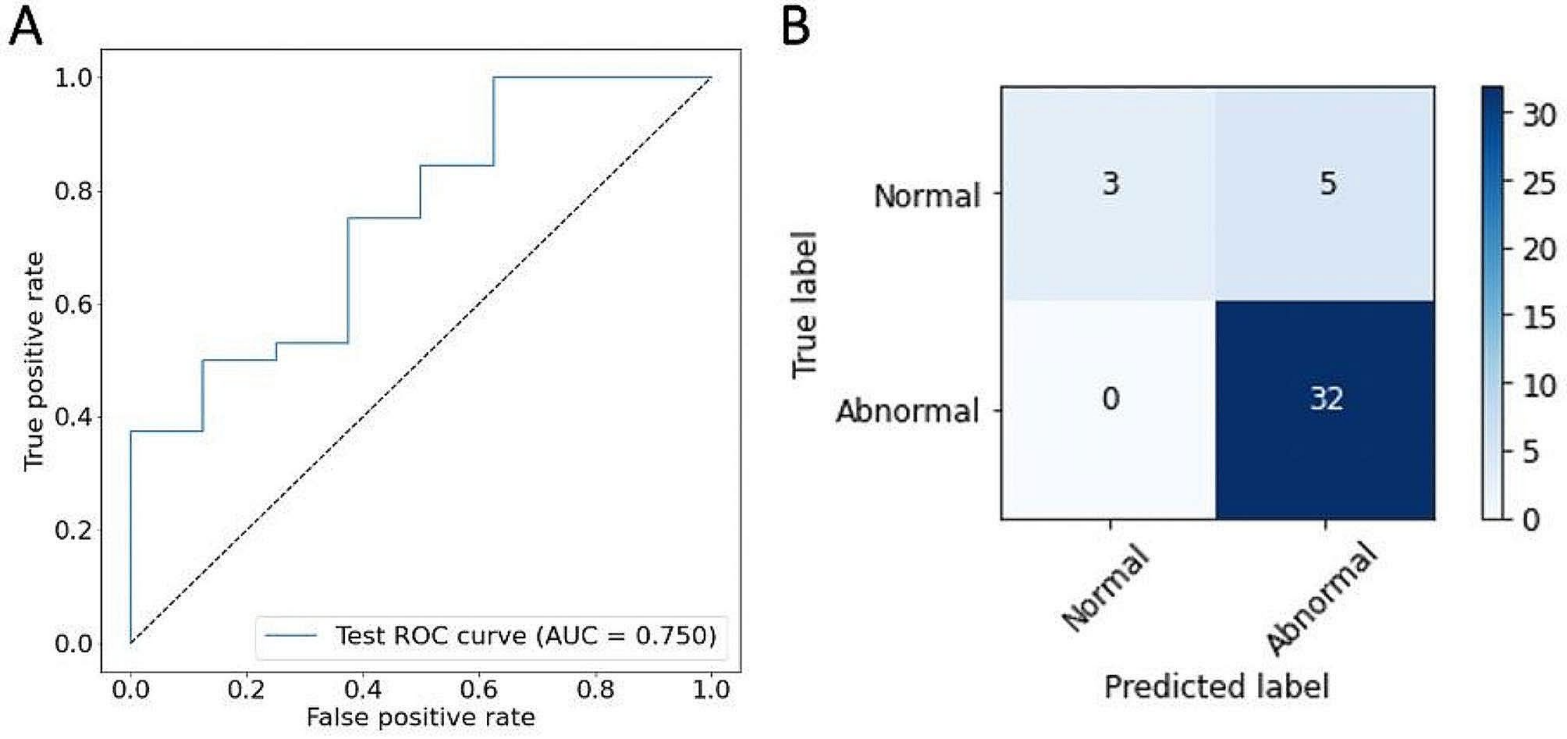
Performance of the model on the PSIR test set for classification as normal or abnormal. **A** shows the ROC curve of the model with an AUC value of 0.75. **B** illustrates the corresponding confusion matrix of the model with an overall accuracy of 88%

**Fig. 3:**
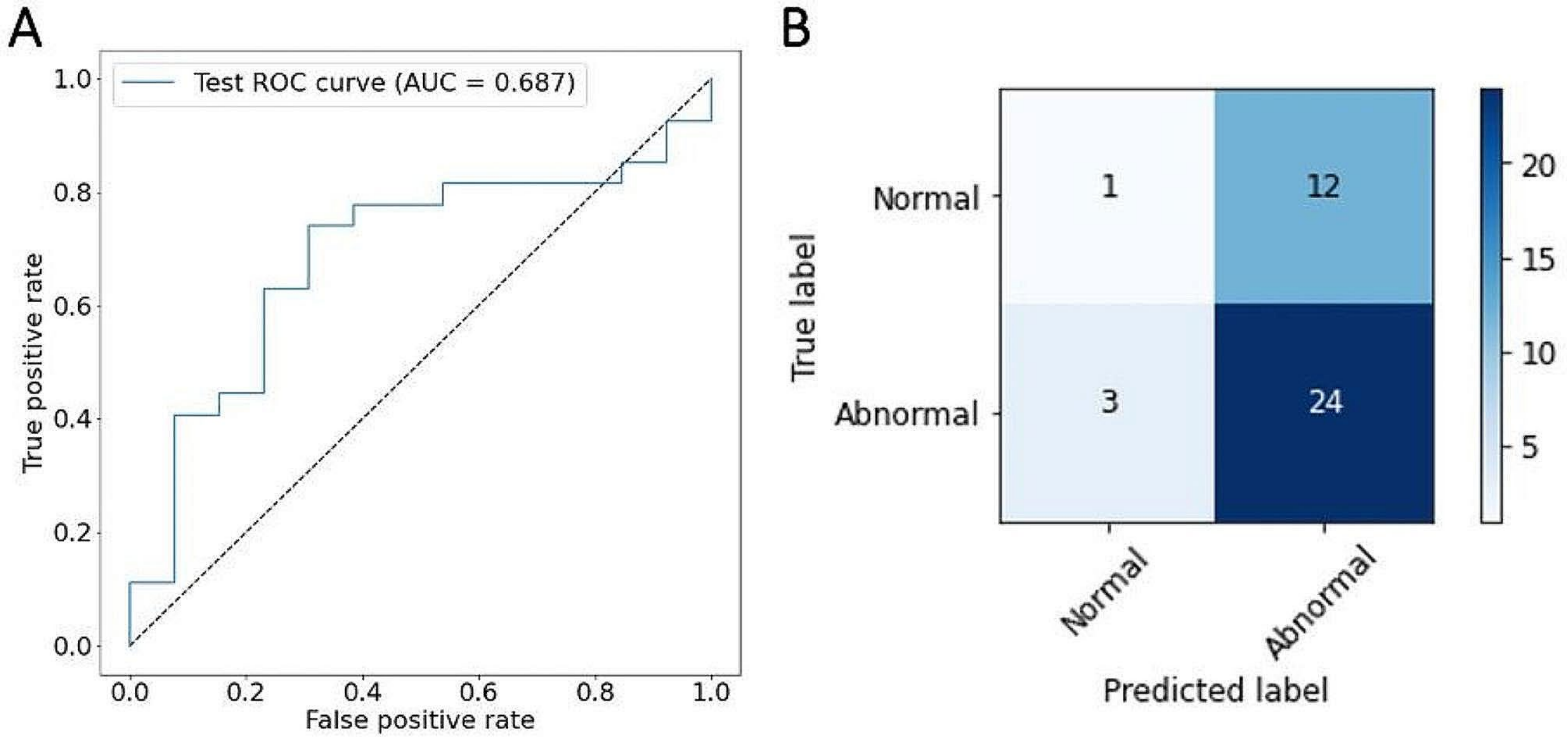
Performance of the model on the T1-mapping test set for classification as normal or abnormal. **A** shows the ROC curve of the model with an AUC value of 0.69. **B** illustrates the corresponding confusion matrix of the model with an overall accuracy of 70%

The ROC curve of the PSIR-based model with its AUC value and the corresponding confusion matrix can be seen in Fig. 2. The ROC curve of the T1 mapping-based model with its AUC value and the corresponding confusion matrix can be seen in Fig. 3.

Thresholds of the right upper corner of the ROC curve of the T1 mapping-based model, where the curve undercuts the diagonal line, classify nearly every case as abnormal, resulting in low specificity. The PSIR-based model, on the other hand, stays above the diagonal line at all times. In the middle part of the ROC graph, the curve of the T1 mapping-based model shows slightly higher sensitivity rates than the PSIR-based model for the same false positive rate (around 0.3). The left section of the ROC diagram, characterized by high specificity, depicts slightly higher sensitivity values for the PSIR-based model.

**Table 6:** Classification performance of both DL models on test sets

Dataset images	Accuracy (%)	Sensitivity (%)	Specificity (%)	FPR (%)	FNG (%)	AUC
PSIR	88	100	38	63	0	0.75
T1 mapping	70	78	54	46	11	0.69

Note. — Dataset refers to the training and evaluation of the two separate DL models on PSIR against on T1 mapping images. FPR stands for false positive rate. FNR stands for false negative rate.

### Visualization of pathology assessment with Grad-CAM

**Fig. 4:**
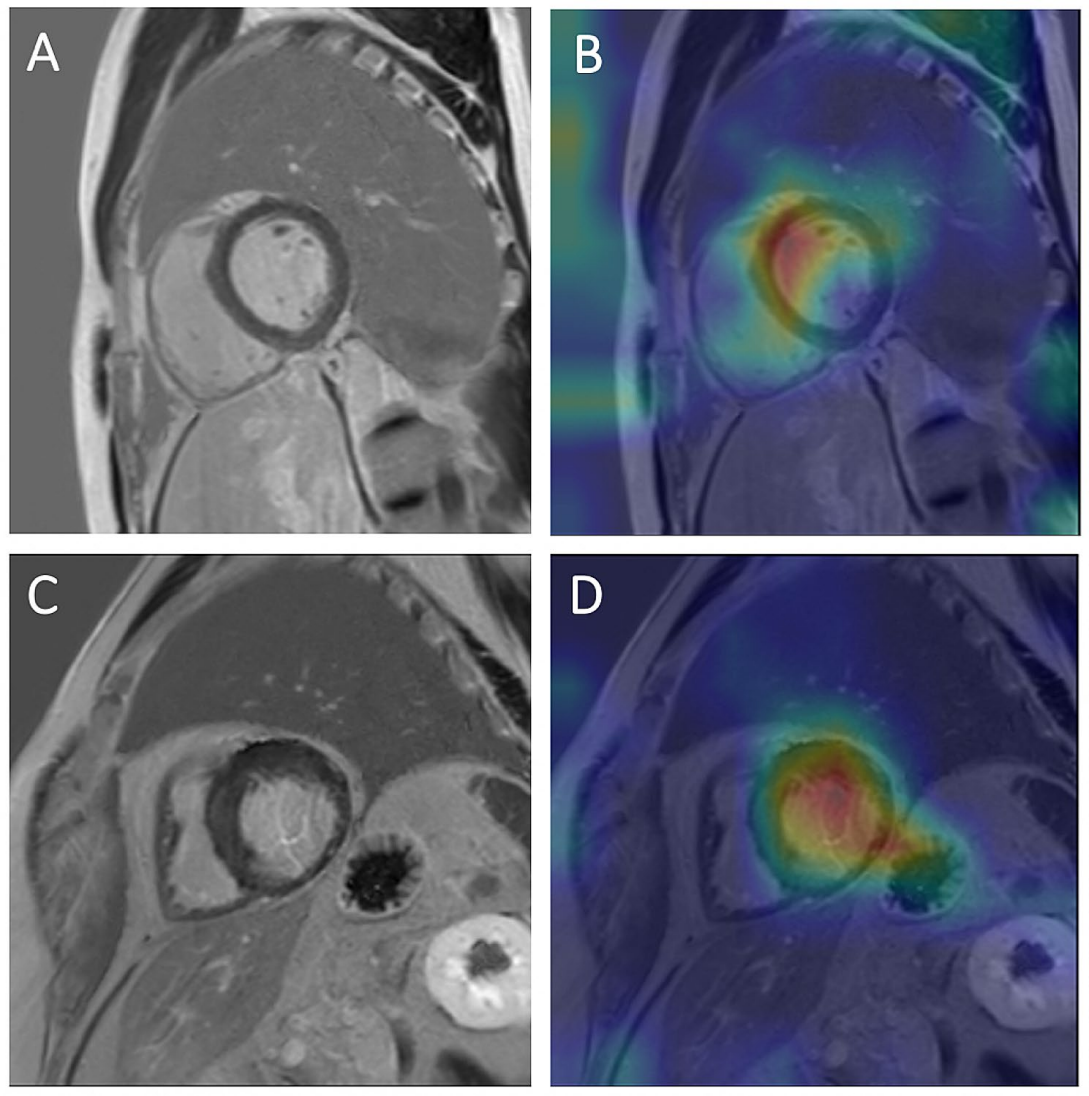
Heatmaps for cardiac pathology assessment on PSIR images. **A, B**: Subject without cardiac pathology. **A** shows the late gadolinium phase sensitive inversion recovery (PSIR) image. **B** shows a heatmap generated by overlaying a gradient-weighted class activation map (Grad-CAM) with the PSIR image. Red indicates higher activation, and blue indicates lower activation. The heatmap shows that the model mainly focused on the myocardial septum for its decision. This was classified by the deep learning model as normal with 86% certainty. **C, D**: Subject with chronic myocardial infarction. **C** shows the late gadolinium phase sensitive inversion recovery (PSIR) image. **D** shows a heatmap generated by overlaying a gradient-weighted class activation map (Grad-CAM) with the PSIR image. Red indicates higher activation, and blue indicates lower activation. The heatmap shows that the model mainly focused on the myocardium of the left ventricle, exhibiting wall thinning and an increase in signal intensity. The deep learning model diagnosed a cardiac pathology with 99% certainty

The Grad-CAMs of the PSIR image samples can be examined in Fig. 4. Two correctly identified examples were chosen for the PSIR-based model, one with and one without cardiac disease. In both cases, the DL network focused its attention on the myocardium and cardiac pathology while assessing the MR images. The heatmaps indicate that our model correctly learned crucial discriminative features for the detection of the heart and classification task. Both examples were classified with high certainty.

**Fig. 5:**
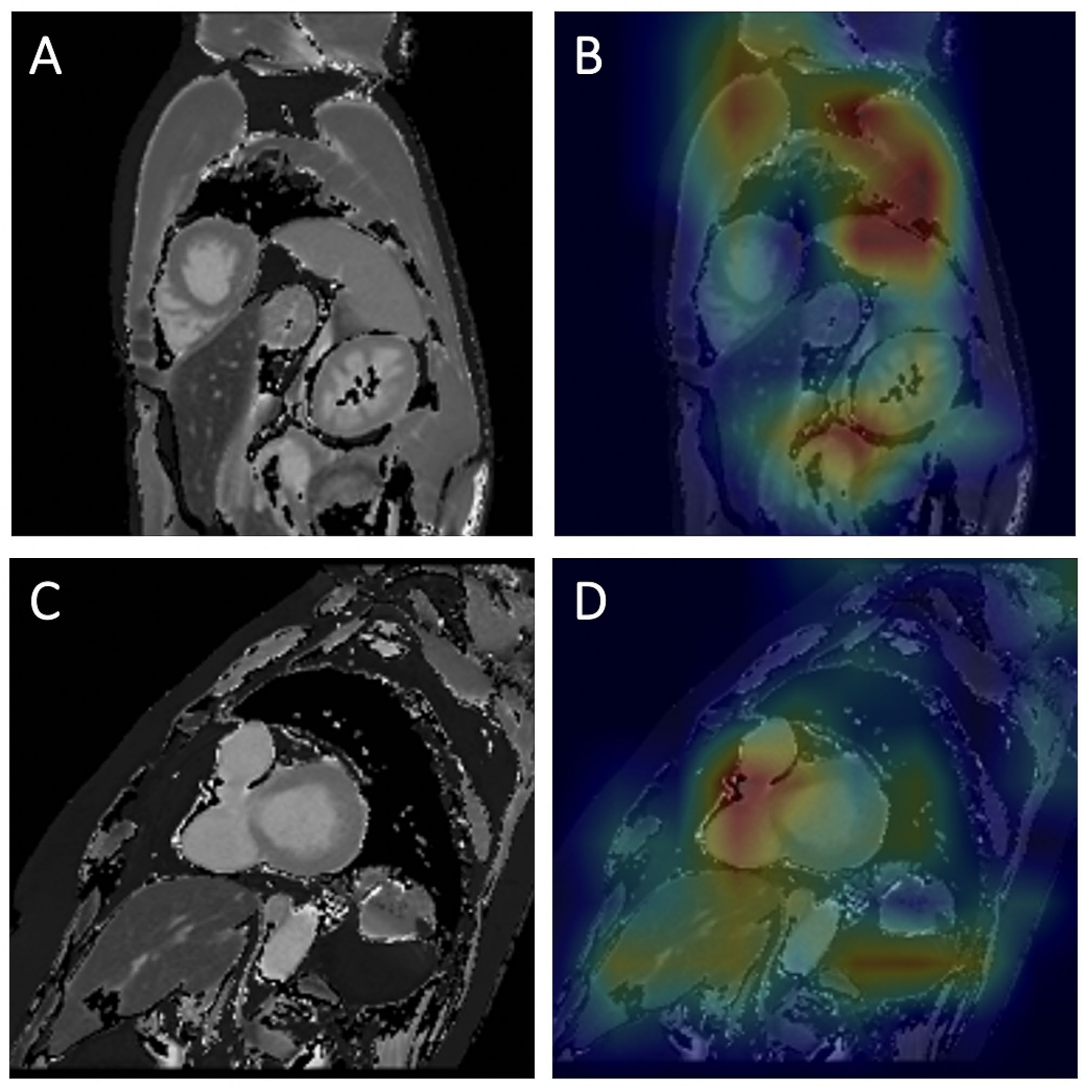
Heatmaps for cardiac pathology assessment on T1 mapping images. **A, B**: Subject without cardiac pathology in the sagittal plane with short axis view. **A** shows the T1 mapping image. In **B**, the image was overlaid with a gradient-weighted class activation map (Grad-CAM), generating a heatmap. The heatmap depicts the focus areas of the model. Red indicates higher activation, and blue indicates lower activation. While making the classification, the network focused on parts of the image other than the heart. Thoracic muscles, spleen, intestines, and lower pole of the kidney represented the focus points. The model classified this case incorrectly as abnormal with 94% certainty. **C, D**: Subject with cardiac disease in the sagittal plane with short axis view. **C** shows the T1 mapping image. In **D**, the image was overlaid with a gradient-weighted class activation map (Grad-CAM), generating a heatmap. The heatmap depicts the focus areas of the model. Red indicates higher activation, and blue indicates lower activation. The strongest focus of the model was the right ventricle, including part of the septum. The kidney and liver represent weaker focus areas of the deep learning model. The network diagnosed a cardiac pathology with 100% certainty

The Grad-CAMs of the T1 mapping image samples can be examined in Fig. 5. Two samples were selected for the T1 mapping-based model. In one example, the network misclassified a subject as abnormal with 94% certainty. The focus points of the DL model for this case were outside the cardiac region. Poorer MR image quality might be a possible reason for the false focus points. The other example, which was correctly labeled as abnormal with 100% certainty, had the right ventricle and septum area as the most important focus points.

## Discussion

### Summary

In this study, we explored the potential of a pretrained DenseNet-161 network to detect pathologies on cardiac MR images. Therefore, we trained and evaluated two DL-based models separately on LGE PSIR and T1 mapping sequences. The aim was to develop an automated assessment tool to differentiate between normal and abnormal myocardium on CMR. Both models showed promising results, reliably recognizing pathologic myocardium with good accuracy of 70% (T1 mapping) and 88% (LGE PSIR). As demonstrated by the Grad-CAMs our models mainly focus on the heart area for their analysis. Adversarial examples near the boundary of the two classes may lead to potential misinterpretation. In addition, misclassification may occur due to poor image quality and artifacts, such as motion or susceptibility distortion. This aspect is in line with lower spatial resolution of T1 mapping compared to LGE PSIR sequence, contributing to its lower accuracy. The disease composition of the abnormal group can also influence the comparative performance between T1 mapping and LGE PSIR.

### Future clinical application of automated cardiovascular disease detection systems as an inline CMR function

CMR is considered the gold standard for cardiovascular assessment with high diagnostic and prognostic value within clinical practice. Its increasing accessibility and integration into clinical routine is of great value for the early detection of cardiac conditions [3, 5, 6].

However, CMR imaging is known for its time-consuming nature, involving lengthy protocols and extended examination times. This time constraint creates logistical challenges. For example, the unavailability of time slots for emergency cases and the delay of critical CMR evaluations that are important for further decision-making and treatment.

Automated disease detection as an inline function in medical imaging could help accelerate the heart assessment process by ruling out normal cases and identifying pathologies even before radiologist review. Focusing on abnormal findings would enable faster diagnosis and intervention. This worklist prioritization optimizes a physician’s time and facilitates a higher volume of scanned patients. Hence, allowing more people to benefit from the examination and receive crucial treatment on time.

In particular, inexperienced physicians can benefit from a reference finding in their decision-making process. Thus, an automated CMR inline detection function offers the potential for more streamlined clinical workflow and enhanced patient care. In low-volume CMR centers, an AI tool aids in diagnosing rare cardiac diseases, in need of profound expert knowledge. This noninvasive approach helps prevent misdiagnosis and ensures accurate and early evidence-based treatment. A time-saving approach that increases patient throughput is especially relevant in populations with a low pretest probability of heart diseases where many healthy subjects need to be ruled out. The deployment of automated tools, such as CNNs, emerges as a promising solution for more efficient screening procedures.

In summary, we aimed to address the aspect of limited availability of expertise in cardiac MRI. Automated assistance by the AI tool as an CMR inline function may be beneficial. Therefore, we did not focus solely on a single pathology. However, a subsequent project could delve into the precise detection of specific cardiac diseases.

### Limitations and future outlook

First, our DL model is constrained to the information entailed in the CMR image. Including additional data from patients’ health records or further test results may enhance future diagnostic performance as they reflect a more holistic approach pursued in daily clinical practice. Second, we extracted our images from consecutive examinations to come close to the prevalence of cases experienced during a daily clinical routine of a university hospital. However, the resulting unbalanced dataset may include underrepresented pathologies showing low sensitivity. Therefore, more training examples of those cases are necessary to increase their classification sensitivity. We emphasize that our analysis covers different pathologic myocardial entities, contributing to a more realistic setting in contrast to numerous studies focusing on one specific pathology and comparing it to healthy volunteers, resulting in stronger thresholds that are much lower. Thus, this approach holds significance for clinical applications and paves the way for future projects addressing related challenges in cardiac imaging. Third, the high discrepancy between sensitivity and specificity is noteworthy. This may be caused due to the unbalanced dataset and the chosen thresholds. The inclusion of different pathologic myocardial entities contributes to a weaker threshold compared to studies concentrating on a single pathology. The chosen dataset and thresholds align with our goal of testing under a real-world clinical setting for an immediate clinical application. The trade-off between high sensitivity and low specificity may yield false positives but may overlook only minor findings. For instance, the PSIR-based model shows a 0% false-negative rate, while the T1 mapping-based model demonstrates an 11% false-negative rate. Given the study’s goal of implementing AI as a CMR inline function in clinical routines, our clinical application envisions AI detecting pathologies before a physician’s review. This strategy, despite leading to a higher false positive rate, prioritizes a low false-negative rate. This prioritization aims to enhance worklist management for examining radiologists. Forth, our dataset of 200 patients contained a diverse setting of patients with cardiac diseases. However, a larger sample size may improve our models’ performances and generalization capability. Fifth, we performed a retrospective study using imaging data from a single center and a single MRI vendor. The subsequent step to enhance our networks involves conducting external multicenter validation on separate larger datasets for a comprehensive evaluation of our models’ robustness and generalization. Finally, the applicability of our networks on different MRI sequences remains uncertain. A potential future project could involve evaluating or training our model on additional sequences to address the complexity of CMRs. Based on our findings we expect that our AI tool has the potential to save time and streamline workflow in clinical routine with minimal costs. A prospective project involving practical clinical implementation on a patient test group would be a future study. This would enable an assessment of the actual time saved, identification of potential oversights, and an exact understanding of the implications for subsequent evidence-based treatments.

Our achieved classification accuracies of 70% and 88% show that the overview of a radiologist is necessary, as we cannot yet fully rely on the DL models. Therefore, final confirmation by a physician remains obligatory at this point in time.

## Conclusion

Two DL-based models using the DenseNet-161 algorithm were separately trained to automatically assess LGE PSIR and T1 mapping cardiac MRIs, showing promising diagnostic performance. Both DL models reliably detected cardiac pathologies and accurately distinguished between normal and abnormal myocardium. The network evaluating T1-mapping images obtained 70% accuracy, and the model based on LGE PSIR images presented 88% accuracy. Routine implementation of DL as an inline function of CMR scanners might streamline diagnostic workflow.

## Data Availability

The data underlying this article will be made available to other researchers upon reasonable request. Please contact us at aleksandra.paciorek@tum.de.
